# Necrostatin-1 enhances the resolution of inflammation by specifically inducing neutrophil apoptosis

**DOI:** 10.18632/oncotarget.8346

**Published:** 2016-03-24

**Authors:** Hongyu Jie, Yi He, Xuechan Huang, Qingyou Zhou, Yanping Han, Xing Li, Yongkun Bai, Erwei Sun

**Affiliations:** ^1^ Department of Rheumatology and Immunology, The Third Affiliated Hospital of Southern Medical University, Guangzhou, Guangdong, China; ^2^ Institute of Clinical Immunology, Academy of Orthopedics, Guangzhou, Guangdong, China; ^3^ Hospital of South China Normal University, Guangzhou, Guangdong, China

**Keywords:** neutrophil, apoptosis, inflammation, Necrostatin-1

## Abstract

Neutrophils play a central role in innate immunity and are rapidly recruited to sites of infection and injury. Neutrophil apoptosis is essential for the successful resolution of inflammation. Necrostatin-1 (Nec-1,methyl-thiohydantoin-tryptophan (MTH-Trp)), is a potent and specific inhibitor of necroptosis[1] (a newly identified type of cell death representing a form of programmed necrosis or regulated non apoptotic cell death) by inhibiting the receptor interacting protein 1(RIP1) kinase. Here we report that Nec-1 specifically induces caspase-dependent neutrophils apoptosis and overrides powerful anti-apoptosis signaling from survival factors such as GM-CSF and LPS. We showed that Nec-1 markedly enhanced the resolution of established neutrophil-dependent inflammation in LPS-induced acute lung injury in mice. We also provided evidence that Nec-1 promoted apoptosis by reducing the expression of the anti-apoptotic protein Mcl-1 and increasing the expression of pro-apoptotic protein Bax. Thus, Nec-1 is not only an inhibitor of necroptosis, but also a promoter of apoptosis, of neutrophils, enhancing the resolution of established inflammation by inducing apoptosis of inflammatory cells. Our results suggest that Nec-1 may have potential roles for the treatment of diseases with increased or persistent inflammatory responses.

## INTRODUCTION

Neutrophils are the most abundant circulating leukocytes in humans. They are key players in the innate immune response and rapidly recruited to sites of infection and injury [2]. However, the defense mechanisms that are powerful in eliminating and digesting invading microorganisms are potentially injurious to surrounding healthy tissues [3]. Thus, it is very important that neutrophils be rapidly and efficiently removed from the inflammatory sites as soon as the invading microorganisms have been cleared away. Neutrophil apoptosis is the major route through which the host maintains homeostasis. This might explain why neutrophils, under steady-state conditions, have the shortest life span among leukocytes in the circulation and in tissues [4, 5].

Neutrophil apoptosis is tightly regulated by a complex network of signaling pathways that control the expression and degradation of the anti-apoptotic protein myeloid cell leukemia 1 proteins (Mcl-1) [6] and the pro-apoptotic Bcl-2 family members Bax and Bad [7], as well as the activation of caspase family [8]. As we know, the apoptotic death of neutrophils is very important for the resolution of inflammation. Unlike necrotic neutrophils, the apoptotic cell membrane remains integrated, thus preventing the release of damage-associated molecular patterns (DAMPs), and the aggravation of inflammatory responses. Furthermore, in the early course of apoptosis, neutrophils express signals to recruit macrophages [5]. Efficient phagocytosis of apoptotic neutrophils by macrophages not only prevents the secondary necrosis of apoptotic neutrophils, and the release of neutrophil debris and pro-inflammatory signals, but also makes macrophages turn into anti-inflammatory cells by inducing the production of cytokines IL-10 and TGF-β [9].

The balance between neutrophil survival and apoptosis is exquisitely regulated by signals from other cells and the inflammatory microenvironment. Inflammatory mediators such as GM-CSF, lipopolysaccharide (LPS) [7], peptidoglycans, CpG DNA, and the acute-phase proteins such as modified C-reactive protein and serum amyloid A (SAA) delay neutrophil apoptosis. Dysfunction in neutrophil apoptosis machinery is considered as critical for the pathogenesis of many chronic human inflammatory diseases, such as cardiovascular diseases [10], acute respiratory distress syndrome[11], rheumatoid arthritis [12] and sepsis [13]. Therefore, searching for novel molecules that can reverse the dysfunction in neutrophil apoptosis machinery may constitute a novel therapy for severe and sustained inflammation and its associated diseases.

Nec-1 is an inhibitor of the receptor interacting protein 1(RIP1) kinase that has usually been used as a potent and specific inhibitor of necroptosis [14, 15]. Encouraging results have been obtained by blocking RIP1 kinase activity with Nec-1 in various experimental disease models, such as ischemia-reperfusion injury in brain [14], heart [16]and kidney [17], systemic inflammatory response syndrome [18], sepsis and retinal cell death [17].

During our investigation of how Nec-1 influences the manner of neutrophil death, inconsistent with the reported conception that Nec-1had no effect on apoptosis[14], we first unexpectedly found that Nec-1 specifically induced neutrophil apoptosis *in vitro*. We also showed that this apoptosis inducing effect was neutrophil specific. And its signaling mechanism may be through down regulation of Mcl-1 and enhancement of Bax expression. Furthermore, we demonstrated *in vivo* that Nec-1 could markedly enhance the resolution of inflammation in a mouse model of LPS-induced acute lung injury. These findings suggest that the resolution of inflammation may be successfully achieved by inducing neutrophil apoptosis *in vivo*, which provides a novel therapeutic strategy to promote resolution of inflammation in many infectious and autoimmune diseases, especially those with severe and sustained, inflammatory disorders.

## RESULTS

### Nec-1 specifically induced human neutrophil apoptosis

**Figure 1:**
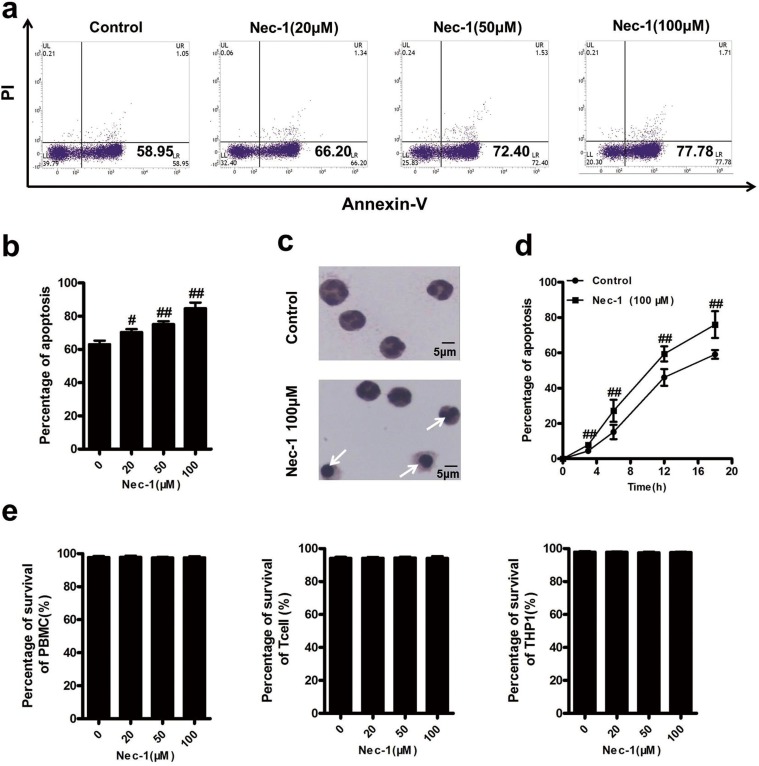
Nec-1 specifically induced human neutrophil apoptosis (**a**, **b**) Nec-1 concentration-dependently induced apoptosis of neutrophils. Human neutrophils (5×10^6^ cells/ml) were incubated for 20h with increasing concentrations of Nec-1. Apoptosis was assessed by Annexin-V–FITC /PI labelling. Data represent the mean ± s.e.m. (n ≥3, ^#^*P* ≤0.05, ^##^*P* ≤0.01 versus control). (**c**) Human neutrophils (5×10^6^ cells/ml) were incubated for 12 h with 100μM Nec-1. In control group, typical neutrophil polymorphic nuclei were seen while in Nec-1 group, condensed nuclei, a typically apoptotic neutrophil morphological change (arrow) were found. (**d**) Nec-1 time-dependently induced apoptosis of neutrophils. Human neutrophils (5×10^6^ cells/ml) were incubated for 3h, 6h, 12 h or 18 h with 100μM Nec-1. Apoptosis was assessed as in Figure 1a (n ≥3, ^##^*P* ≤0.01 versus control). (**e**) Nec-1 had no apoptotic inducing effect on PBMC, T cells and THP1 cells. Cells (5×10^6^ cells/ml) were incubated for 20h with increasing concentrations of Nec-1(^##^*P* ≥0.05 versus control).

In the study of the effect of Nec-1 on neutrophil death, we isolated neutrophils from human peripheral blood through density gradient centrifugation. Isolated human neutrophils were incubated with Nec-1 over 20h [14, 19, 20]. Although our initial attempt was to investigate the effect of Nec-1 on neutrophil necrosis, we unexpectedly found that Nec-1 could induce neutrophil apoptosis (Figure 1a, 1b). Compared with no treatment control (63.15±5.15%), neutrophils treated with 20μM (70.44±4.43%), 50μM (75.19±4.28%) and 100μM (84.78±8.36%) of Nec-1 for 20h displayed marked increase in apoptotic rates (*P*<0.05, control vs for different concentration groups) by flow cytometry analysis with PI/Annexin V staining. And Nec-1 induced human neutrophil apoptosis could also be identified morphologically by their condensed nuclears and membrane blebing compared with control group (Figure 1c).In a time-course study (Figure 1d), Nec-1 (100μM) increased the rates of apoptosis time dependently. Most interestingly, Nec-1 had no effect on peripheral blood mononuclear cell (PBMC), T cells and THP1 cells (Figure 1e), indicating that Nec-1 specifically promotes the apoptosis of neutrophils.

**Figure 2:**
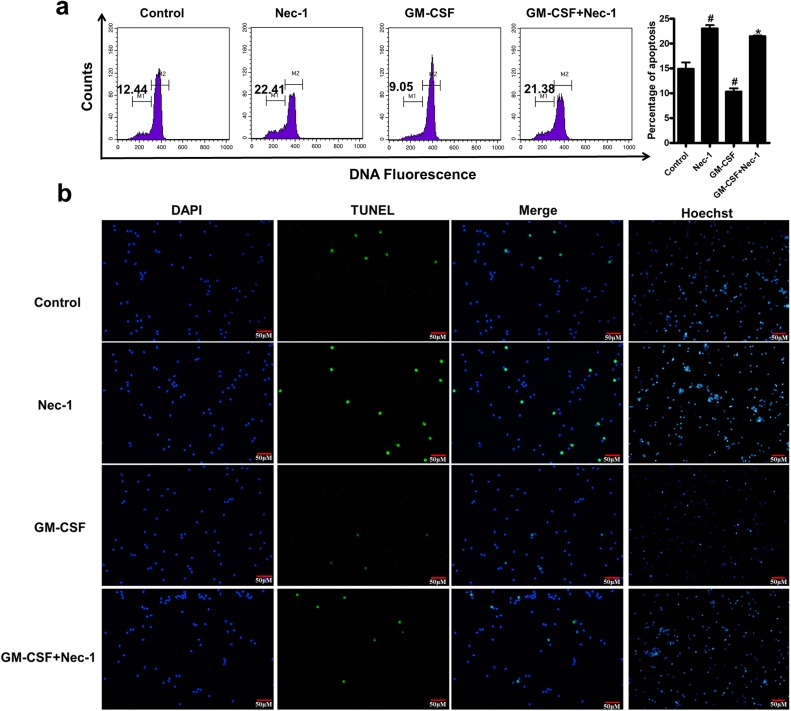
Nec-1 induced human neutrophil apoptosis was further confirmed by analyzing the assay of TUNEL, sub-G1 hypodiploid and nuclear morphology of neutrophil Human neutrophils (5×10^6^cells/ml) were incubated for 16h with Nec-1 (100μM), GM-CSF(20ng/ml). (**a**) Neutrophils were stained with PI and analyzed for hypodiploid cells by flow cytometry. Hypodiploid population was assessed as in Figure 2a (^#^*P* ≤0.05 versus control, *P ≤0.05 versus GM-CSF alone group). (**b**) Neutrophil apoptosis was examined by TUNEL assay. Compared with the control the number of TUNEL positive cells were increased in the Nec-1 (100μM) treatment groups (Figure 2b). (**c**) Nuclear morphology was examined with membrane-permeable blue Hoechst. Compared with control group, more hypercondensed (brightly stained) neutrophils were found in the group of treated with Nec-1 (Figure 2b).

Apoptosis was further confirmed by analyzing sub-G1 hypodiploid, the assay of terminal deoxyribonucleotidyl transferase-mediated dUTP-digoxigenin nick end-labelling (TUNEL) and morphology. Nec-1 treated neutrophils were stained with PI and analyzed for hypodiploid cells by flow cytometry. The apoptosis of neutrophils was identified in a DNA histogram. Compared with control group, neutrophils treated with Nec-1 for 16h showed more hypodiploid population (*P*<0.05, Figure 2a). For TUNEL assay, neutrophils were incubated for 16h with Nec-1, compared with the control the number of TUNEL positive cells were increased in the Nec-1 (100μM) treatment groups(Figure 2b). Nec-1 induced neutrophils apoptosis was also confirmed by analyzing the nuclear morphology with membrane-permeable blue Hoechst. Compared with control group, more hypercondensed (brightly stained) neutrophils were found in the group of treated with Nec-1 for 16h (Figure 2b). Therefore, Nec-1 induced the apoptosis of neutrophils.

### Nec-1 overrode the anti-apoptosis effect by some survival factors

**Figure 3:**
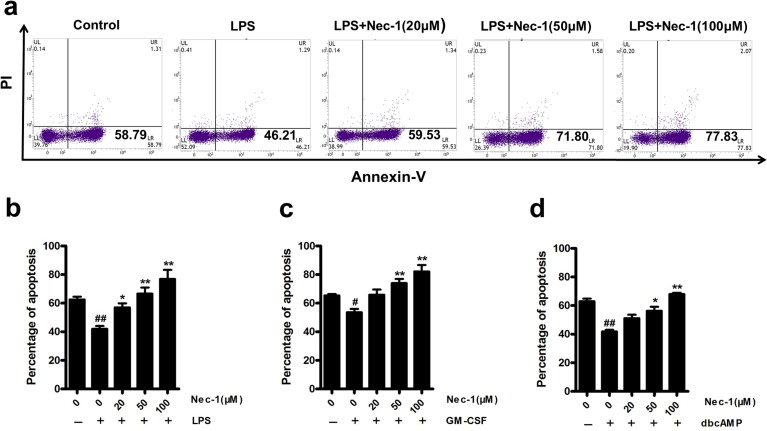
Nec-1 reversed the inhibition of apoptosis induction (**a**, **b**) Nec-1 reversed LPS-mediated inhibition of neutrophil apoptosis. Human neutrophils (5×10^6^ cells/ml) were pre-incubated with 100 ng/ml of LPS for 30 min. Increasing concentrations of Nec-1 were then added and the cells incubated for another 20h. Apoptosis was assessed as in Figure 1a (n≥3, ^##^*P* ≤0.01 versus control, *P ≤0.05, **P ≤0.01 versus LPS alone group). (**c**) Nec-1 reversed GM-CSF-mediated inhibition of neutrophil apoptosis. Human neutrophils (5×10^6^ cells/ml) were pre-incubated with 20ng/ml of GM-CSF alone for 30 min. Increasing concentrations of Nec-1 were then added and the cells incubated for another 20 h. Apoptosis was assessed as in Figure 1a (n≥3, ^#^*P* ≤0.05 versus control, **P ≤0.01 versus GM-CSF alone group). (**d**) Nec-1 reversed dbcAMP -mediated inhibition of neutrophil apoptosis. Human neutrophils (5×10^6^ cells/ml) were pre-incubated with 0.1mM of dbcAMP for 30 min. Increasing concentrations of Nec-1 were then added and the cells incubated for another 20 h. Apoptosis was assessed as in Figure 1a (n≥3, ^##^*P* ≤0.01 versus control, *P ≤0.05, **P ≤0.01versus dbcAMP alone group).

In acute or chronic inflammation, neutrophil apoptosis is usually blocked by microbial products or endogenous danger signal such as LPS, GM-CSF and dbcAMP (dibutyryl-cAMP) [21], which may result in sustained and uncontrollable inflammation and tissue damage. So we next examined the effect of Nec-1 on the function of these anti-apoptotic molecules on neutrophil apoptosis. As shown in Figure 3a-3d), Nec-1 overrode all of these above survival signals in a concentration dependent manner.

### Nec-1 induced neutrophil apoptosis is caspase-dependent

**Figure 4:**
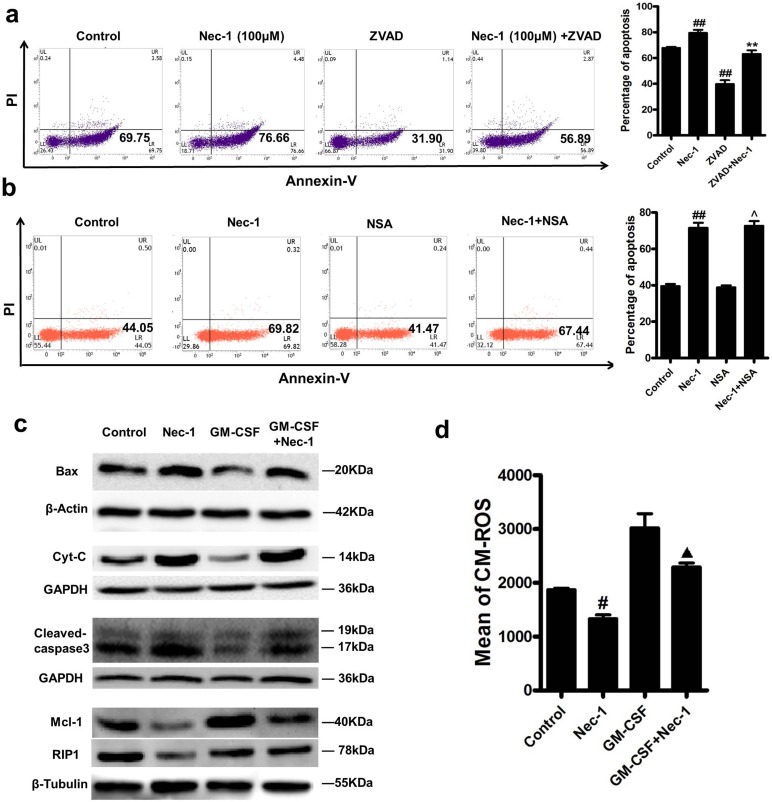
Nec-1 mediated neutrophil apoptosis was caspase-dependent, with reduced neutrophil Mcl-1 expression and up regulated expression of Bax and cleaved caspase-3 (**a**) Human neutrophils (5×10^6^cells/ml) were incubated for 20h with Nec-1 (100μM) with or without zVAD-fmk (50μM). Apoptosis was assessed as in Figure 4a (n≥3, ^##^*P* ≤0.01 versus control, **P ≤0.01versus Nec-1 alone group). (**b**) Human neutrophils (5×10^6^cells/ml) were incubated for 16h with 100μM Nec-1 with or without 0.5μM Necrosulfonamide(NSA), Apoptosis was assessed as in Figure 4b (n≥3, ^##^*P* ≤0.01 versus control, ^*P* >0.05 versus Nec-1 alone group). (**c**) Human neutrophils (5×10^6^ cells/ml) were treated with buffer control, Nec-1 (100μM), GM-CSF (20 ng/ml), or GM-CSF (20 ng/ml) plus Nec-1 (100μM) for 12h. After cell lysis, proteins were subjected to Mcl-1, Bax, Cyt-C, cleaved caspase-3 and RIP1. Blots are representative of at least three separate experiments. (**d**) Detection of mitochondrial membrane potential. Human neutrophils (5×10^6^ cells/ml) were treated with buffer control, Nec-1 (100μM), GM-CSF (20 ng/ml), or GM-CSF (20 ng/ml) plus Nec-1 (100μM) for 16h. Human neutrophils were stained with 250 nM of MitoTracker Red CMXRos, and analyzed by flow cytometry. Mitochondrial membrane potential was assessed as in Figure 4d (n≥3, ^#^*P* ≤0.05 versus control, ^▲^*P* ≤0.05 versus GM-CSF group).

In order to elucidate the mechanisms by which Nec-1 promotes neutrophil apoptosis in un-stimulated state or in the presence of anti-apoptotic signals, we further investigated the roles of caspases in this process. Incubating neutrophils with the pan-caspase inhibitor zVAD-fmk prevented Nec-1–induced apoptosis (Figure 4a), indicating the apoptosis induction is caspase dependent. Importantly, our results showed that Nec-1 resulted in caspase-3 (CPP-32, Apoptain, Yama, SCA-1) cleavage in neutrophils, a step that has been proved critical for caspase-3 activation and execution of apoptosis [22-24], (Figure 4c). Furthermore, although a pro-survival factor GM-CSF could reduce the caspas-3 cleavage, Nec-1-treatment again fully blocked the effects of GM-CSF on the expression of cleaved caspase-3.

### Nec-1 reduced neutrophil Mcl-1 expression, while increased Bax and induced the release of cytochrome C from mitochondria and loss of mitochondrial potential

Neutrophil apoptosis is controlled by a complex network of signaling pathways that regulate the key molecules involved in apoptosis and survival, including the anti-apoptotic protein myeloid cell leukemia 1 (Mcl-1) and the pro-apoptotic Bcl-2 family member Bax. The anti-apoptotic protein Mcl-1 has been proved to be essential for the survival of neutrophils [6], while the pro-apoptotic Bax is a main driver of neutrophil apoptosis[7].

Western blot analysis was performed to examine the expression of Mcl-1 and Bax. Mcl-1 was up regulated by GM-CSF treatment, which was blocked by Nec-1(Figure 4c). On the contrary, the decreased expression of Bax by GM-CSF was also blocked by Nec-1 treatment (Figure 4c). The results indicate that a shift from survival factor Mcl-1 to apoptotic factor Bax plays a key role in the mechanisms of Nec-1 induced neutrophil apoptosis.

Also, we found the loss of mitochondrial potential as a result of Nec-1 treatment. Neutrophils were stained with MitoTracker Red CMXRos followed by FACS analysis to exam the mitochondrial potential. The expression of CMXRos in neutrophils treated with Nec-1 for 16h showed was less than the control group (*P*<0.05, Figure 4d). Furthermore, the release of cytochrome C from mitochondria was assessed by Western blotting. Compared with the control group, more release of cytochrome C was found in Nec-1 treatment group. These results indicate that Nec-1 induced the loss of mitochondrial potential.

### Nec-1 induced neutrophils apoptosis was not coupled to inhibited necroptosis and possibly related to the inhibition of RIP1 expression

Necrosulfonamide(one kind of RIP3 inhibitors) was used to block cell necroptosis. We found that Nec-1 promoted neutrophil apoptosis could not be inhibited by necrosulfonamide (Figure 4b) as there was no significant differences in neutrophil apoptosis between the Nec-1group and the Necrosulfonamide plus Nec-1 group(*P*<0.05).

Although it has been reported that RIP1 has an important role in inducing apoptosis [25].We also found that Nec-1 inhibited the expression of RIP1(Figure 4c), implying that Nec-1 induced neutrophils apoptosis may not be related to RIP1 pro-apoptotic signialing.

### Nec-1 promoted resolution of inflammation by inducing neutrophil apoptosis *in vivo*

As dysfunction in neutrophil apoptosis machinery plays a crucial role in ongoing inflammation and our *in vitro* studies showed that neutrophil apoptosis was markedly and specifically induced by Nec-1, we next investigated the effects of Nec-1 on the resolution of neutrophil mediated inflammation *in vivo*. LPS-induced acute lung injury model is typified by disruption of neutrophil apoptosis machinery [26]. Thus, we used LPS-induced acute lung injury mouse model to assess the effects of the Nec-1 on inflammatory cell recruitment and to investigate inflammatory mechanisms.

**Figure 5:**
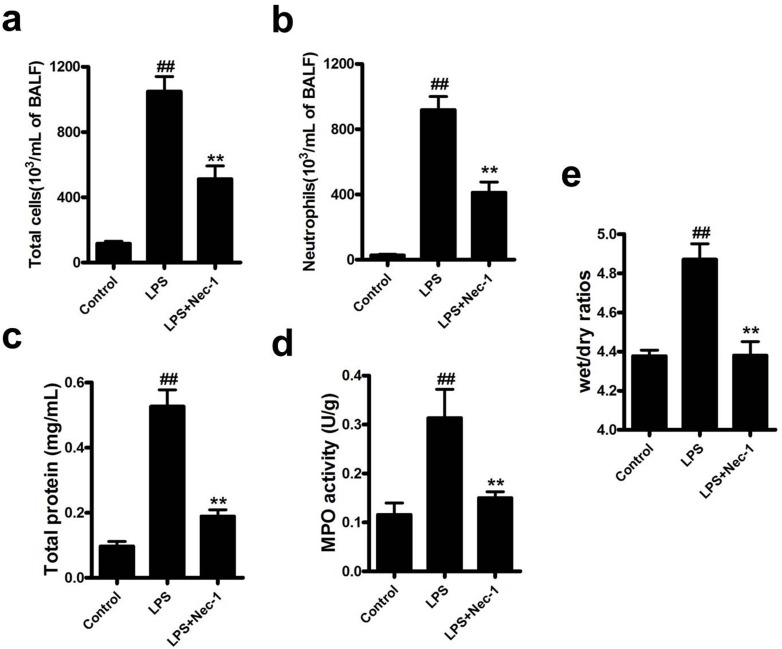
Nec-1 inhibited cellular infiltration in BAL of LPS-challenged mice and attenuates LPS-induced acute lung injury LPS (5μg/50μl PBS) was dripped into the nares of mice. Nec-1(5mg/kg) was injected intraperitoneallly 6h before LPS challenge. Control mice received equal volumes of PBS or DMSO instead of LPS or Nec-1, respectively. Mice were killed 4h after LPS instillation and BAL was collected. The numbers of total cells and neutrophils in BAL were examined. All values are mean±s.em (n =6). (**a**, **b**) Nec-1 reduced the numbers of total cells and neutrophils in BAL of LPS-challenged mice (^##^*P* ≤0.01 versus control. **P ≤0.01 versus LPS alone group). (**c**) Nec-1 diminished LPS-induced increase in total protein concentration. Mice were killed 4h after LPS instillation and BAL collected, and total protein concentration in BAL examined. All values are mean ± s.e.m. (n =6, ^##^*P* ≤0.01 versus control. **P ≤0.01versus LPS alone group). (**d**) Nec-1 diminished LPS-induced increase in lung MPO activity. Mice were killed 4h after LPS instillation and the lungs removed. Lung tissues were homogenized in PBS, and the homogenate assayed for MPO activity. All values are mean ± s.e.m. (n =6, ^##^*P* ≤0.01 versus control. **P ≤0.01versus LPS alone group). (**e**) Nec-1diminished LPS-induced increases in lung wet/dry ratios. Lung wet/dry ratios were determined as described in Materials and Methods. All values are mean ± s.e.m. (n =6, ^##^*P* ≤0.01 versus control. **P ≤0.01versus LPS alone group).

**Figure 6:**
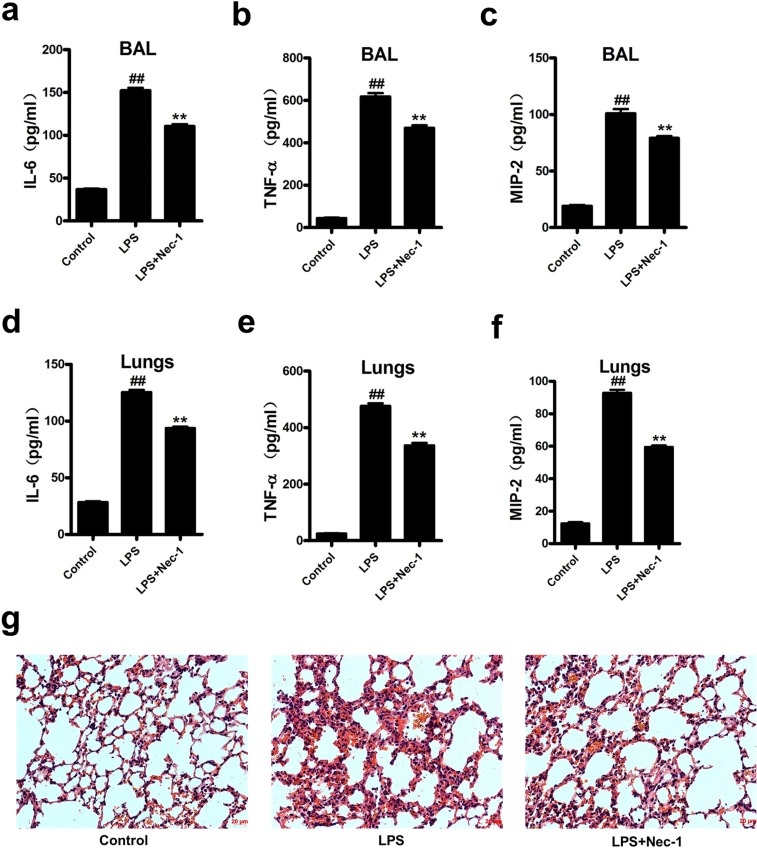
Nec-1 ameliorated LPS-induced inflammatory changes LPS (5μg/50μl PBS) was administered into the nares of mice. Nec-1(5mg/kg) was injected intraperitoneallly 6h before LPS challenge. Control mice received equal volumes of PBS or DMSO instead of LPS or Nec-1, respectively. (**a-c**) Mice were killed 4h after LPS instillation and BAL collected. Levels of IL-6, TNF-α and MIP-2 in BAL were determined by ELISA. All values are mean ± s.e.m. (n =6, ^##^*P* ≤0.01 versus control. **P ≤0.01versus LPS alone group). (**d-f**) Mice were killed 4h after LPS instillation and lungs were removed. Levels of IL-6, TNF-α and MIP-2 in lung homogenates were determined by ELISA. All values are mean ± s.e.m. (n =6, ^##^*P* ≤0.01 versus control. **P ≤0.01versus LPS alone group). (**g**) Nec-1 ameliorated LPS-induced inflammation in lung tissues. 4h after LPS challenge, the lungs were fixed, embedded in paraffin. After H&E staining, histological examination was performed by light microscopy (magnification ×200).

**Figure 7:**
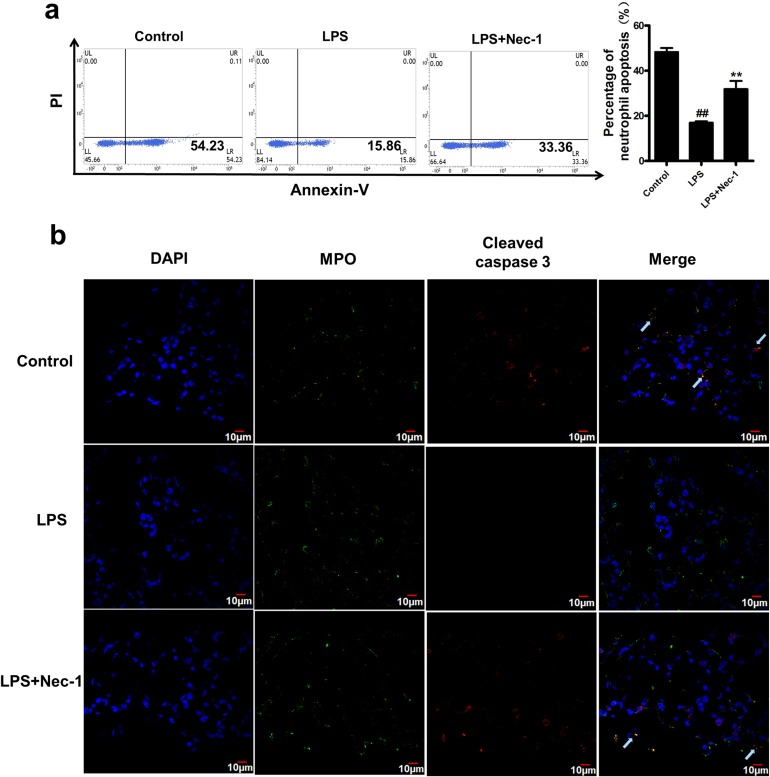
Nec-1 reversed LPS induced inhibition of neutrophil apoptosis in the BAL and lung LPS (5μg/50μl PBS) was administered into the nares mice. Nec-1(5mg/kg) was injected intraperitoneallly 6h before LPS challenge. Control mice received equal volumes of PBS or DMSO instead of LPS or Nec-1, respectively. (**a**) Neutrophils apoptosis were assessed by flow cytometry, Ly6G/Ly6C (Gr-1) and CD11b double positive staining was used to label mouse neutrophil. Nec-1 significantly reversed the inhibition of neutrophil apoptosis in the BAL fluid compared with the LPS alone group (^##^*P* ≤0.01 versus control. **P ≤0.01versus LPS alone group). (**b**) Mice were killed 4h after LPS instillation and the lungs were removed and kept in 4% paraformaldehyde for 4h, then immersed in 30% sucrose at 4°C for 3–4 days. Then 15μm sections were cut on a cryostat. Myeloperoxidase(MPO) was used to mark neutrophils in the lungs and cleaved caspase-3 was used to mark apoptosis cells in the lungs. Sections were visualized with confocal laser scanning system. Arrow indicates MPO and cleaved caspase-3 double positive staining, which was used to label the apoptosis of mouse neutrophil. Nec-1 inhibited neutrophil infiltration and induced neutrophil apoptosis in the lungs of LPS-challenged mice compared with the LPS alone group.

Assessment of the inflammatory cells in bronchoalveolar lavage (BAL) showed that Nec-1 reduced the total inflammatory cells as well as neutrophils by more than 50% as compared with that in LPS group (Figure 5a, 5b). Similarly, total protein concentrations in BAL, an indicator of lung leakage, and pulmonary myeloperoxidase (MPO) activity, a marker of neutrophil infiltration into the lungs, were also dramatically reduced in Nec-1-treated mice (Figure 5c, 5d). Wet/dry ratios, a measure of interstitial pulmonary edema and severity of acute lung injury (ALI), was diminished in Nec-1-treated mice as compared with LPS treated mice (Figure 5e). Furthermore, the release of the pro-inflammatory mediators such as IL-6, TNF-α, and MIP-2 in the BAL and lung homogenates were also significantly decreased by Nec-1(Figure 6a-6f). Histological examination of the lungs also showed that Nec-1 markedly reduced the inflammation in the lungs of LPS-challenged mice as compared with the LPS alone group (Figure 6g). In order to know whether Nec-1 reduced the inflammation was mediated by the induction of neutrophil apoptosis, we assessed neutrophil apoptosis in the BAL. The results showed that apoptosis was significantly blocked by LPS treatment and Nec-1 could significantly induced neutrophil apoptosis in the BAL (Figure 7a). Immunohistochemistry of the lungs also indicated that Nec-1 inhibited neutrophil infiltration and induced neutrophil apoptosis in the lungs of LPS-challenged mice compared with the LPS alone group (MPO used to mark neutrophils in the lungs and cleaved caspase-3 used to mark apoptosis cells in the lungs) (Figure 7b).

Overall, our results indicate that Nec-1 promotes the resolution of lung inflammation by accelerating neutrophil apoptosis in LPS treated mice.

## DISCUSSION

Neutrophils play a central role in inflammatory responses to infection in that they release toxic ingredients to effectively kill invading microorganisms. These toxic molecules, however, are also able to cause tissue damage if the inflammatory responses are not tightly controlled. Therefore, the inflammatory responses induced by neutrophils must be regulated with exquisite precision and timing [27]. Apoptosis, a programmed cell death that maintains membrane integrity and retains toxic neutrophil contents, is one of such regulatory mechanisms to limit and resolve excessive inflammatory responses [28]. Apoptosis and efficient phagocytosis of apoptotic cells have been recognized to play a critical role in the resolution of inflammation [29]. Anti-inflammatory effects of apoptotic cells have been demonstrated in many inflammatory disease models [9, 21, 30, 31]. The benefits of apoptotic neutrophils are double as phagocytosis of them shifts macrophages from a pro-inflammatory state into an anti-inflammatory state, which further restrains the inappropriate inflammatory responses. Dysfunction in neutrophil apoptosis machinery will accelerate inflammatory responses, damage surrounding tissues and cause a variety of diseases. Thus, normal neutrophil apoptosis is very important for the control of tissue inflammation.

Nec-1 was identified following the screening of a chemical library of some 15,000 compounds for inhibitors of necroptosis induced by TNF-α in the presence of zVAD.fmk. The chemical was found to selectively target the kinase activity of RIP1, a key mediator of necroptosis. Nec-1 has been used widely both *in vitro* and *in vivo* to study the role of necroptosis [32]. Obviously, Nec-1 was found to inhibit necroptosis by activating death-domain receptor(DR) in the presence of caspase inhibitors in all previously described cellular models of necroptosis [33]. Nec-1 did not induce Jurkat cells apoptosis that was evidenced by the observation that apoptotic rate detected by Annexin V/PI staining, as well as characteristic apoptotic morphology [14], such as cellular shrinkage and chromatin condensation, was not changed by Nec-1 treatment. Many studies on Nec-1 were mainly concerned with its effect on necroptosis [20, 34], while few studies were carried out about its effects on apoptosis. Weidong Han found that Nec-1 reverts shikonin-induced necroptosis to apoptosis in HL60 cells [35], this research give us a hint that Nec-1 may have an effect on apoptosis.

Like other researchers, we were to study neutrophil necroptosis with Nec-1. Unexpectedly, we found that Nec-1 could specifically induce neutrophil apoptosis *in vitro*. This finding promoted us to further investigate the mechanisms and significances of Nec-1 induced neutrophil apoptosis. Our results revealed that Nec-1 induced caspase-dependent apoptosis in a time and concentration dependent manner. Interestingly, Nec-1 induced apoptosis even in the presence of various powerful pro-survival agents (GM-CSF, LPS and dbcAMP) that inhibit human neutrophil apoptosis through different molecular mechanisms. For example, GM-CSF suppresses neutrophil apoptosis by activating PI3K and MEK1-ERK1/2 pathways, LPS by activating PI3K and NF-κB pathways, and dbcAMP by activating PKA pathways. Thus, the ability of Nec-1 to override endogenous pro-survival factors further suggests its potential application in inflammatory diseases where concentrations of such mediators are massively increased. Most importantly, we also found that Nec-1 did not influence apoptosis of peripheral blood mononuclear cells, suggesting that Nec-1 specifically induces neutrophil apoptosis. This finding is of pivotal importance in that we may be able specifically promote neutrophil apoptosis without affecting the survival of other blood cells.

Neutrophil apoptosis can be induced by multiple mechanisms. Mcl-1 is a key Bcl-2 family protein located in both the nucleus and cytoplasm of neutrophils [36, 37]. Many studies have evidenced that various stimuli able to regulate Mcl-1expression finally induce or inhibit neutrophil apoptosis can [38-42]. In addition, mice with a conditional knockout of Mcl-1 in neutrophils showed severe defects in neutrophil survival due to increased apoptosis [6]. More recently, it has been shown that murine neutrophil numbers are reduced because of apoptosis during differentiation, if Mcl-1 is knocked out in the myeloid lineage [43]. These data suggest that Mcl-1 plays an indispensable role in promoting the survival of neutrophils. In contrast to Mcl-1, Bax is a Bcl-2-family member that promotes neutrophil apoptosis [44], and interacts with the mitochondria, and reduces neutrophil survival [45, 46]. Our results have demonstrated that Nec-1-induced apoptosis is associated with a down regulation of Mcl-1 expression and up regulation of Bax, and is caspase dependent. In addition, we also found that caspase 3 cleavage were activated by Nec-1 treatment. These results may explain why Nec-1 induces human neutrophil apoptosis and override various pro-survival factors.

Next we tried to evaluate whether our *in vitro* finding that induction of neutrophil apoptosis by Nec-1would have anti-inflammatory effects *in vivo*. To this end, we used a model of LPS-induced acute lung injury, a well-established acute inflammation model, to assess the effects of the Nec-1 on lung inflammation. Inhibition of neutrophil apoptosis has been demonstrated in LPS-induced acute lung injury, and those neutrophils accumulated in the lungs produce high levels of proinflammatory mediators, including cytokines, chemokines, and reactive oxygen species [47, 48]. We have now shown that Nec-1 greatly diminished inflammation and lung injury in LPS-treated mice. Nec-1 not only reduced the infiltration of total inflammatory cells, such as neutrophils as well as monocytes/macrophages, but also inhibited the release of pro-inflammatory cytokines, including TNF-α, IL-6 and MIP-2. In addition, pulmonary MPO expression [48] (a marker of neutrophil infiltration into the lungs), lung wet/dry ratios (a measure of interstitial pulmonary edema and severity of acute lung injury), protein concentrations in BAL (an indicator of lung leak), were all significantly diminished in Nec-1-treated mice. Histological examination also showed that Nec-1 reduced LPS-induced inflammation in acute lung injury. Furthermore, the apoptosis of neutrophil in BAL and the lungs was induced by Nec-1, providing further evidence that the anti-inflammatory mechanism of the Nec-1 is due to the promotion of neutrophil apoptosis.

Therefore, our *in vitro* and *in vivo* work has demonstrated that Nec-1 promotes the resolution of inflammation by inducing neutrophil apoptosis. As mentioned, Nec-1 has already been proven to be of benefit in models of ischemia–reperfusion injury, mostly attributed to its role as a necroptosis inhibitor [14, 34, 49]. As neutrophil infiltration is most common and plays a very important role in ischemia–reperfusion injury [50-52]. Our results suggest the effect of Nec-1 treatment in ischemia reperfusion models may not only result from the inhibition of necrosis but also due to the promotion of neutrophil apoptosis. Similarly, suppressed neutrophil apoptosis has been detected in sepsis [53, 54] and promoting neutrophil apoptosis may be a cure [13].

**Figure 8:**
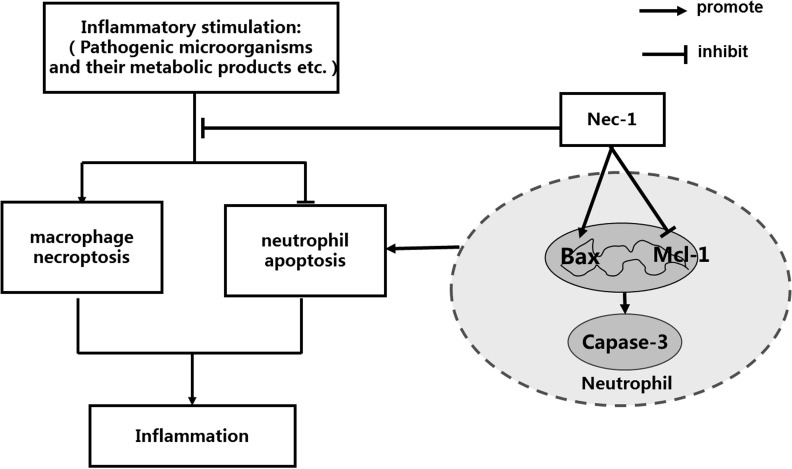
Schematic illustration for the effects Nec-1 on neutrophil apoptosis and macrophage necroptosis, underlying the pivotal roles of Nec-l in the treatment of inflammatory disorders Pathogenic microorganisms and their metabolic products, for example LPS, TNF-α can induce necroptosis in macrophage, which promotes the production of inflammation. At the same time, pathogenic microorganisms and their metabolic products, for example LPS, IL-1and IL-6 inhibit apoptosis in neutrophil, which also amplify the production of inflammatory responses. On the one hand, Nec-1 enhances the resolution of inflammation by inhibiting necroptosis of macrophage, and on the other hand, Nec-1 reduces inflammation by inducing neutrophil apoptosis. The unique effect of Nec-1 to promote neutrophil apoptosis and inhibit necroptosis in other cells enables Nec-1 to be a very powerful inflammation inhibitor that may have potential clinical values in treating servere or sustained inflammatory disorders.

In conclusion, we found that Nec-1 specifically induced human neutrophil apoptosis by down regulating neutrophil Mcl-1 expression as well as promoting Bax expression. And the Nec-1 induced apoptosis of neutrophils is caspase dependent and caspase-3 cleavage plays a key role. Furthermore, we also found that Nec-1 promoted the resolution of inflammation in LPS-induced acute lung injury by inducing neutrophil apoptosis. As Nec-1 has been found to inhibit necroptosis in other cells, our study suggests that Nec-1 enhances the resolution of inflammation by inducing neutrophil apoptosis, as well as by inhibiting necroptosis in macrophages [55] and surrounding tissue cells (Figure 8). Further investigation to compare the mechanisms orchestrating apoptosis signaling pathways in neutrophils and necroptosis pathways in other tissue cells might reveal novel targets for the manipulation of tissue inflammation. At the same time, the unique effects of Nec-1 to specifically promote neutrophil apoptosis and inhibit necroptosis in other cells enables Nec-1 to be a very powerful inflammation inhibitor, that may have potential clinical values in treating severe or sustained inflammatory disorders.

## MATERIALS AND METHODS

This study was approved by the Ethics Committee of the Third Affiliated Hospital of Southern Medical University. Trial Registration clinicaltrials.gov Identifier: NCT02385331.

### Neutrophil isolation and assessment of apoptosis

Human blood neutrophils were isolated by dextran sedimentation and centrifugation as described [56], and the cells (2.5×10^6^/ml,37°C) cultured in RPMI 1640 medium containing 10% autologous serum. The cells were incubated with the following reagents, Nec-1(Calbiochem), db-cAMP (Sigma), GM-CSF (Sigma), LPS (Escherichia coli O111:B4, Sigma), and Necrosulfonamide (NSA, Tocris Bioscience). Apoptosis was assessed with with various methods, such as double staining with FITC-labeled Annexin-V in combination with PI (BD Biosciences), Terminal deoxynucleotidyl transferase dUTP nick end labeling (TUNEL) assay, PI staing for sub-G1 hypodiploid, Hochest staining and microscopy used to confirm the presence of morphological characteristic of apoptosis [57, 58]. TUNEL assay Human neutrophils (5×10^6^ cells/ml) were incubated for 16h with Nec-1, GM-CSF, and then fixed in 4% paraformaldehyde for the TUNEL assay,using an in situ detection kit (Roche, Indianapolis, USA) according to the manu-facturer's instructions. Cells were counterstained with 4′,6-diamidino-2-Phenylindole (DAPI) to visualize all of the nuclei. Stained cells were viewed under a fluorescence microscope.

Propidium iodide (PI) staining for apoptosis. Human neutrophils (5×10^6^ cells/ml) were incubated with Nec-1as well as GM-CSF. After 16 h, cells were harvested and washed in cold PBS, fixed with 70 % ethanol at −4°C for 4 h, and then were stained with propidium iodide (PI). DNA contents were analyzed using flow cytometry.

Analysis of nuclear morphology. Human neutrophils (5×10^6^ cells/ml) were incubated with Necrostatin-1, GM-CSF. After 16 h, cells were fixed with methanolacetic acid for 10 min followed by staining with Hoechst 33258 (Sigama) staining at room temperature at dark for 5 min. The cells were then washed twice with PBS, examined and immediately photographed under a fluorescence micro-scope. Apoptotic cells weredefined on the basis of nuclear morphology changes such as chromatin condensation and fragmentation.

MitoTracker For MitoTracker staining, human neutrophils were treated with 250 nM of MitoTracker Red CMXRos (Invitrogen) in culture medium for 30 min at 37°C. The cells were fixed, washed, and analyzed by flow cytometry.

### Western blotting

Neutrophils (5×10^6^) were lysed with ice-cold RIPA(Radio Immunoprecipitation Assay) buffer containing a protease inhibitor cocktail for 30 min before centrifugation(14,000g, 4°C, 15 min). All manipulations were performed on ice. Protein samples (30μg per lane) were resolved by SDS-PAGE and then transferred to polyvinylidene difluoride (PVDF) membranes. Blots were blocked with 5% skimmed milk powder in TBS plus Tween before probing with antibodies to cleaved caspase-3, Mcl-1, Bax, Cyt-C, RIP1,β-Tubulin, GAPDH (Cell Signaling Technologies) andβ-actin (EarthOx, LLC).

Mice. Male C57BL/6J mice (aged 8–12 weeks, weighed 18–22 g) were obtained from Experimental Animal Center of Southern Medical University (Guangzhou, China). The animal experiments were conducted according to the ethical guidelines for animal experiments of Sun Yat-sen University and under experimental license approved by Guangdong, China.

### Mouse models of acute lung injury

Animals were anesthetized with pentobarbital sodium. Then, LPS (5μg/50μl PBS, Escherichia coli O111:B4, Sigma) was dripped into the nares [59]. Nec-1(5mg/kg) was injected intraperitoneallly 6h before LPS challenge. Control mice received equal volumes of PBS and DMSO instead of LPS and Nec-1, respectively. Four hours after LPS challenge, mice were anaesthetized and then killed by exsanguination.

### Bronchoalveolar lavage collection and cell count

The mice were anesthetized and euthanized. The trachea was exposed and a 20-gauge angiocatheter inserted. The lungs were lavaged with three separate 0.5ml ice-cold PBS. The broncho-alveolar lavage (BAL) was centrifuged at 400 × g for 10 min at 4°C, and the supernatant stored at −80°C for the assessment of cytokines and total proteins. The cells were re-suspended in 200 μl of cold PBS, and the cell types determined by examining 200 cells on the Wright–Giemsa-stained smears. The neutrophil apoptosis was assessed by flow cytometry, Ly6G/Ly6C (Gr-1) (BD Biosciences) and CD11b (BD Biosciences). Gr-1 and CD11b double positive staining was used to label mouse neutrophil [60]. Mice prepared for histological experiments did not undergo bronchoalveolar lavage to keep tissue integrity.

### Immunohistochemistry

The lungs were removed and kept in 4% paraformaldehyde for 4 h, then immersed in 30% sucrose at 4°C for 3–4 days. Then 15-μm sections were cut on a cryostat. The primary antibodies were rabbit anti-cleaved caspase-3 (Asp175) (1:500, Cell Signaling Technology), mouse anti-Myeloperoxidase [2D4] (ab90810) (1:100, Abcam). Sections were visualized with confocal laser scanning system (Leica DM IRE2, Germany). MPO was used to mark neutrophils in the lungs and cleaved caspase-3 used to mark apoptosis cell in the lungs. MPO and cleaved caspase-3 double positive staining cells were recognized as the apoptotic mouse neutrophils.

### Measurement of pro-inflammatory cytokines in bronchoalveolar lavage and lungs

The levels of TNF-α, IL-6 and MIP-2 were measured with ELISA kits according to the manufacturer's protocol (R&D Systems, USA).

### Measurement of MPO

Lung tissues were frozen in liquid nitrogen and then homogenized in PBS. The homogenate was used to determine MPO according to the manufacturer's instruction (NanjingJianCheng Bioengineering Institute, China)

### Total protein concentrations in BAL

Total protein concentration in the supernatant of BAL was quantified by the Bradford method with bovine serum albumin (Beyotime Institute of Biotechnology, China) as a standard.

### Wet/dry lung weight ratios

All mice prepared for determining lung wet/weight ratios were of identical ages. Lungs were excised, rinsed briefly in PBS, blotted, and then weighed to obtain the “wet” weight. Lungs were then dried in an oven at 80°C for 24h to obtain the “dry” weight.

### Statistical analysis

Data are expressed in term of mean±S.E.M. Statistical analysis was performed by a one-way ANOVA test and values of *P* <0.05 were considered significant.

## Abbreviations

Nec-1: Necrostatin-1
GM-CSF: Granulocyte-macrophage colony-stimulating factor
LPS: Lipopolysaccharide
RIP1: Receptor- interacting protein 1
Mcl-1: myeloid cell leukemia 1
DAMPs: damage-associated molecular patterns
Bcl-2: B-cell lymphoma-2
IL-10: interleukin-10
TGF-β: transforming growth factor-β
SAA: serum amyloid A
PBMC: peripheral blood mononuclear cell
dbcAMP: dibutyryl-cAMP
BAL: bronchoalveolar lavage
MPO: Myeloperoxidase
ALI: acute lung injury
IL-6: interleukin-6
TNF-α: Tumor Necrosis Factor
MIP-2: macrophage inflammatory protein-2
DR: death-domain receptor
NF-κB: nuclear factor κB
PI3K: phosphatidylinositol 3-kinase
PKA: protein kinase A
PVDF: polyvinylidene difluoride
TUNEL: Terminal deoxynucleotidyl transferase dUTP nick end labeling
NSA: Necrosulfonamide
C(Cyt-C): cytochrome
